# Initial antibiotic selection and microbiological recurrence in treated bacteriuria caused by low-risk AmpC-harboring Enterobacterales: *serratia marcescens, Morganella morganii,* and *Providencia* spp.

**DOI:** 10.1017/ash.2026.10908

**Published:** 2026-10-08

**Authors:** Yingsi Fang, Matthew Gwiazdon, Ryan Chapin, Christopher McCoy, Kendall Bell

**Affiliations:** 1 Beth Israel Deaconess Medical Center, USA; 2 Beth Israel Lahey Health, USA

## Abstract

**Objective:**

Despite updated guidance supporting antimicrobial susceptibility testing-guided antibiotic selection for low-risk AmpC-harboring organisms (*Serratia marcescens*, *Morganella morganii*, and *Providencia* spp.), broad-spectrum AmpC-stable β-lactams remain commonly used. We aimed to compare microbiological recurrence after initial treatment with AmpC-hydrolysis-susceptible β-lactams (AmpC-vulnerable) versus AmpC-stable β-lactams or non-β-lactam agents.

**Design:**

This single-center retrospective cohort included 112 adults treated for urinary isolates of the three organisms between October 2019 and June 2024, including 13 with asymptomatic bacteriuria. Microbiological recurrence was assessed at 28 and 365 days after therapy completion.

**Setting:**

An academic medical center.

**Results:**

Among patients initially receiving AmpC-vulnerable β-lactams versus AmpC-stable β-lactams or non-β-lactam agents, 28-day recurrence occurred in 2/79 (2.5%) versus 3/33 (9.1%), respectively; 365-day recurrence occurred in 5/79 (6.3%) versus 4/33 (12.1%).

**Conclusions:**

These findings provide preliminary evidence relevant to susceptibility-guided treatment of low-risk AmpC organisms in patients with urinary tract infections (UTIs) or bacteriuria. Interpretation is limited by few events, treatment crossover, and a culture-based outcome.

## Introduction

Historically, “SPACE-M” organisms (eg, *Serratia*, *Providencia*/*Pseudomonas*, *Acinetobacter*/indole-positive *Proteus*, *Citrobacter*, *Enterobacter*, and *Morganella*) were considered at risk for inducible AmpC production, prompting broader therapy than indicated by antimicrobial susceptibility testing (AST).^1^ Subsequent evidence identified species-specific risk, particularly for *Enterobacter cloacae* complex, *Citrobacter freundii*, and *Klebsiella aerogenes*.^2–4^ New educational mnemonics such as “HECK Yes” reflect this evolving understanding which includes *Hafnia alvei*, *E. cloacae*, *C. freundii*, *K. aerogenes*, and *Yersinia enterocolitica*.^5^ The 2024 and 2026 IDSA guidance supports AST-guided antibiotic selection for *Serratia marcescens*, *Morganella morganii*, and *Providencia* spp., including susceptible agents vulnerable to AmpC hydrolysis, such as ceftriaxone (CRO).^4,6^

Despite updated guidance, broad-spectrum AmpC-stable β-lactams, primarily cefepime (FEP) and carbapenems, remain commonly used.^7^ UTIs are an important stewardship target because of high prescribing volume, while shorter courses and high urinary β-lactam concentrations may limit the clinical impact of emergent resistance.^4^ Building on existing literature in bacteremia of low-risk AmpC producers,^8^ we compared microbiological recurrence after initial AmpC-hydrolysis-susceptible β-lactams (AmpC-vulnerable) versus AmpC-stable β-lactams or non-β-lactams for urinary isolates of these organisms. This pooling reflects relative AmpC stability of FEP/carbapenems and the absence of AmpC hydrolysis of fluoroquinolones (FQ) and trimethoprim–sulfamethoxazole (TMP/SMX), without assuming equivalent spectrum or clinical effects.^4,6^

## Methods

This single-center retrospective cohort included non-pregnant adults aged ≥18 years receiving ≥48 hours of active antibiotics for urinary isolates of *S. marcescens*, *M. morganii*, or *Providencia* spp. between October 2019 and June 2024. Patients were identified from a microbiology isolate repository using a first-isolate-per-patient-per-year approach similar to that used for antibiogram preparation. The institutional antibiogram was used to identify organisms of interest. All eligible patients identified from available repository records during the study period were included as a convenience sample, and no predetermined sample-size target or a priori sample-size calculation was used. Exclusions were antibiotic treatment for other infectious syndromes, including bacteremia; polymicrobial cultures requiring broader Gram-negative or additional Gram-positive coverage; and target isolates intermediate or resistant to CRO or piperacillin–tazobactam (TZP). Other polymicrobial cultures were retained without organism-number or colony-count thresholds. The study protocol was approved by the Institutional Review Board of Beth Israel Deaconess Medical Center (BIDMC; 2024P000842) and was conducted in accordance with the principles of the Declaration of Helsinki. Individual informed consent was waived due to the retrospective nature of the study. Data were obtained through manual review of the electronic medical record and entered and managed using REDCap hosted at BIDMC.

Group 1 (AmpC-vulnerable β-lactams) comprised initial treatment with CRO, cefpodoxime (CPD), and TZP. Group 2 (AmpC-stable β-lactams or non-β-lactam agents) comprised FEP, meropenem (MEM), TMP/SMX and FQ. The index regimen was the first active agent administered for ≥48 hours, and brief empiric exposures of less than 48 hours preceding the index regimen did not determine assignment. For example, one dose of FEP followed by at least 48 hours of CRO would have resulted in assignment to Group 1. Subsequent regimens were summarized separately and did not change assignment.

Patients documented as having asymptomatic bacteriuria were retained if they received antibiotics at the treating providers’ discretion. Urinary and nonurinary symptoms were classified based on provider documentation with overlapping categories; nonspecific presentations were not independently adjudicated to determine whether symptoms were attributable to a UTI. Complicated UTI was classified using historical criteria, including male sex, urinary catheter or nephrostomy tube, urinary obstruction, structural or functional urinary tract abnormalities, diabetes, urinary retention, and immunocompromise (Table 1).^9–12^ These criteria differ from the 2025 IDSA approach, which emphasizes infection extending beyond the bladder with systemic illness or upper tract findings and includes catheter-associated UTIs. Male sex, underlying urologic abnormalities, diabetes, or immunocompromise alone no longer establishes complicated UTI. However, patients with complicated UTIs may still have underlying conditions such as having an indwelling urinary catheter, neurogenic bladder, urinary obstruction, or urinary retention.^13^ The historical classification was therefore retained because available documentation did not permit reliable retrospective assessment of symptom attribution, tenderness location, and catheter-associated symptoms.

**Table 1.** Baseline characteristics Table 1 long description.

	Group 1^a^ AmpC- vulnerable β-lactams n = 79	Group 2^a^ AmpC-stable β-lactams/non-β-lactams n = 33	Total cohort n = 112
**Male sex, n (%)**	49 (62.03)	21 (63.64)	70 (62.50)
**Age, years, median (IQR)**	76 (69–84)	72 (65–76)	75 (66–81)
**Level of care, n (%)**			
ED	19 (24.05)	5 (15.15)	24 (21.43)
Non-ICU floor	57 (72.15)	25 (75.76)	82 (73.21)
ICU	3 (3.80)	3 (9.09)	6 (5.36)
**Hospital length of stay, days, median (IQR)**	8 (2–14)	7 (3–14)	7.5 (2.75–14)
**Clinical signs/symptoms, n (%)**			
**Urinary**			
Dysuria	29 (36.71)	17 (51.52)	46 (41.07)
Frequency	12 (15.19)	2 (6.06)	14 (12.50)
Urgency	9 (11.39)	2 (6.06)	11 (9.82)
Flank pain	7 (8.86)	5 (15.15)	12 (10.71)
Tenderness	5 (6.33)	1 (3.03)	6 (5.36)
Hematuria	2 (2.53)	0 (0.00)	2 (1.79)
**Non-urinary**			
Fever	19 (24.05)	7 (21.21)	26 (23.21)
Hypotension	7 (8.86)	7 (21.21)	14 (12.50)
Leukocytosis	8 (10.13)	5 (15.15)	13 (11.61)
Altered mental status	27 (34.18)	5 (15.15)	32 (28.57)
Others^b^	8 (10.13)	3 (9.09)	11 (9.82)
**Asymptomatic** ^c^	9 (11.39)	4 (12.12)	13 (11.61)
Historical complicated UTI classification, n (%)	61 (77.22)	29 (87.88)	90 (80.36)
Male sex	49/61 (80.33)	21/29 (72.41)	70/90 (77.78)
Indwelling catheter or nephrostomy tube	22/61 (36.07)	15/29 (51.72)	37/90 (41.11)
Obstructive uropathy	10/61 (16.39)	6/29 (20.69)	16/90 (17.78)
Functional or anatomical abnormalities of the urinary tract	7/61 (11.48)	8/29 (27.59)	15/90 (16.67)
Diabetes mellitus	8/61 (13.11)	5/29 (17.24)	13/90 (14.44)
Baseline urinary retention	8/61 (13.11)	2/29 (6.90)	10/90 (11.11)
Immunocompromised state	2/61 (3.28)	2/29 (6.90)	4/90 (4.44)
Cancer on active chemotherapy	1/61 (1.64)	1/29 (3.45)	2/90 (2.22)
Kidney transplant recipient	1/61 (1.64)	1/29 (3.45)	2/90 (2.22)
Others^d^	2/61 (3.28)	1/29 (3.45)	3/90 (3.33)
**Isolated organisms of interest, n (%)**			
*Serratia marcescens*	31 (39.24)	14 (42.42)	45 (40.18)
*Morganella morganii*	26 (32.91)	14 (42.42)	40 (35.71)
*Providencia* spp.	22 (27.85)	5 (15.15)	27 (24.11)
Polymicrobial with co-pathogens	18 (22.78)	12 (36.36)	30 (26.79)
*Escherichia coli*	8	6	14
*Klebsiella *spp*.*	5	4	9
*Proteus mirabilis*	3	2	5
*Enterobacter cloacae*	1	0	1
*Citrobacter freundii*	1	0	1

a Group 1: CRO/CPD/TZP (n = 79). Group 2: FEP/MEM (n = 11) or FQ/TMP-SMX (n = 22), pooled n = 33. These initial-treatment groups do not imply combination therapy or clinical equivalence.

b Other symptoms include falls, syncope, weakness, fatigue, penile pain, purulent suprapubic-catheter discharge, malfunctioning nephrostomy tube, and elevated CRP.

c Recorded asymptomatic bacteriuria as determined by the treating providers.

d Other factors: ileal conduit, preheart transplant, and spinal surgery.

The primary outcome was microbiological recurrence, defined as re-isolation of the same organism in urine culture within 28 days after treatment completion. Secondary outcomes included recurrence of the same isolate within 365 days, antibiotic changes, and treatment duration. Recurrence with retreatment at 28 days was assessed post hoc, however, recurrence symptoms were unavailable. Changes from initial antibiotics were classified as escalation or de-escalation/oral stepdown using study-specific conventions based on antimicrobial spectrum. Intravenous (IV) to oral (PO) switches were included in either category, depending on the agents involved. For example, CRO to CPD and FEP to TMP/SMX were classified as de-escalation, whereas CRO to a fluoroquinolone was classified as escalation because of the broader spectrum of the latter.

Counts, percentages, medians, and interquartile ranges were calculated, and two-sided Fisher’s exact tests were used for outcome comparisons (Python 3.12.13; SciPy 1.17.0). Post hoc analyses excluded asymptomatic bacteriuria, initial non-β-lactam comparators, both, or any documented TMP/SMX or FQ exposure (Table S2). A two-sided *P* value < .05 was considered statistically significant. No adjustments for multiple comparisons were applied. ChatGPT/Codex assisted with analysis code (OpenAI; https://chatgpt.com; 7 September 2026; GPT-6 Astra).

## Results

During the study period, 112 unique patients had a positive urine culture for at least one of the three organisms of interest: 79 in Group 1 (CRO, 60; CPD, 15; TZP, 4) and 33 in Group 2 (FEP, 9; MEM, 2; TMP/SMX, 11; FQ, 11). Overall, 70 (62.5%) were male, the median age was 75 years, and 82 (73.2%) received non-ICU inpatient care (Table 1). Asymptomatic bacteriuria treatment was reported in 9/79 (documented contexts included 1 kidney transplant, 1 pre-heart transplant, 2 had indwelling catheters, 5 unknown) and 4/33 patients (1 indwelling catheter, 3 unknown). Historical complicated UTI criteria were met by 90/112 (80.4%), and 70/90 (77.8%) were male with overlapping risk factors. Polymicrobial cultures occurred in 18/79 (22.8%) and 12/33 (36.4%).

28-day microbiological recurrence occurred in 2/79 (2.5%) versus 3/33 (9.1%) patients (Fisher’s exact test, *P* = .151). 28-day recurrence with retreatment occurred in 2/79 patients in Group 1 and 2/33 in Group 2 (*P* = .580). One additional recurrence in Group 2 was not considered a true infection and therefore was not retreated. 365-day recurrence occurred in 5/79 (6.3%) versus 4/33 (12.1%; *P* = .445; Table 2). Repeat isolates had the same susceptibility pattern (5/5 and 4/4). Antibiotic changes occurred in 40/79 (50.6%) and 10/33 (30.3%) patients, and median total treatment duration was 7 days in both groups (Tables 2 and S1). Post hoc sensitivity analyses are shown in Table S2.

**Table 2.** Primary and Secondary Outcomes Table 2 long description.

	Group 1 AmpC- vulnerable β-lactams n = 79	Group 2 AmpC-stable β-lactams/non-β-lactams n = 33	*P* value (two-sided Fisher’s exact)
**Primary outcome**			
Microbiological recurrence within 28 days of therapy completion, n (%)	2 (2.53)	3 (9.09)	.151
**Secondary outcomes**			
Microbiological recurrence within 365 days of therapy completion, n (%)	5 (6.33)	4 (12.12)	.445
Hospitalization for recurrence	4/5 (80)	4/4 (100)	
Treated for recurrence	4/5 (80)	3/4 (75)	
Isolate with the same susceptibility pattern as prior	5/5 (100)	4/4 (100)	
28-day recurrence with retreatment, n (%)	2 (2.53)	2 (6.06)	.580
**Change from initial antibiotics, n (%)**			
Escalate	12 (15.19)	0 (0)	
Broader spectrum	3	0	
IV to PO	9	0	
De-escalate/oral-stepdown	28 (35.44)	10 (30.30)	
IV to PO	26	6	
Narrower spectrum	2	4	
No change	39 (49.37)	23 (69.70)	
**Antibiotic duration, days, median (IQR)**			
Total duration of antibiotics	7 (5–8.5)	7 (5–7)	
Total duration of active antibiotics	7 (5–8)	7 (5–7)	

## Discussion

Our study focused exclusively on urinary isolates of three species at low risk of clinically significant AmpC overproduction. One retrospective cohort study by Sapozhnikov and colleagues reported similar UTI outcomes but pooled both low- and moderate-risk species.^14^ Another retrospective study by Mulbah and colleagues found similar outcomes with definitive CRO versus AmpC-stable therapy in low-risk AmpC bacteremia; only 37.4% had a genitourinary source.^8^ Our findings extended this evidence to initial antibiotic selection for urinary isolates. Microbiological recurrence rates were numerically lower following initial treatment with AmpC-vulnerable β-lactams than with AmpC-stable β-lactams or non-β-lactam agents, although differences were not statistically significant at either 28 or 365 days after therapy completion. The same susceptibility pattern reported at recurrence provided some reassurance in observed cases, and these findings were consistent with the updated guidance supporting susceptibility-guided prescribing.^4,6^

We acknowledged several limitations. First, retrospective documentation may have missed external recurrences and exact treatment durations. Antibiotic dosing was not collected; therefore, dosing adequacy and its potential association with outcomes could not be assessed. Inclusion of treated asymptomatic bacteriuria and nonspecific presentations also limited interpretation, as microbiological recurrence does not necessarily represent symptomatic infection or clinical failure. Second, only eligible patients’ data were entered into REDCap, so screening records for patients who were not enrolled were not retained. Small event counts, treatment-selection confounding, heterogeneous comparators, and crossover may also have limited interpretation. Third, historical complicated UTI criteria differ from current definitions. Imaging was often unavailable, while incomplete symptom attribution and tenderness documentation prevented reliable retrospective cystitis/pyelonephritis classification. Last, findings cannot be extrapolated to bacteremia secondary to UTIs, which were excluded.

In conclusion, in this population of patients treated for urinary isolates of organisms at low risk of clinically significant AmpC overproduction, microbiological recurrence rates did not differ significantly between initial AmpC-vulnerable β-lactam therapy and AmpC-stable β-lactam or non-β-lactam therapy. These findings provide additional evidence relevant to susceptibility-guided antibiotic selection, although the small number of events limits conclusions regarding comparative efficacy. Larger prospective studies are needed to further evaluate the use of AmpC-vulnerable antibiotics in symptomatic UTIs, particularly in renal transplant recipients and other immunocompromised patients.

## Supporting information

10.1017/ash.2026.10908.sm001 Fang et al. supplementary material Fang et al. supplementary material

## Data Availability

The corresponding author had full access to data in the study and had the final decision to submit the manuscript for publication.

## Supplementary material

The supplementary material for this article can be found at https://doi.org/10.1017/ash.2026.10908

## Table 1. Long description

A table comparing baseline characteristics of patients with AmpC-vulnerable and AmpC-stable beta-lactams. The table has 25 rows and 5 columns. Column headers are Group 1, Group 2, and Total cohort. Group 1 is further divided into AmpC-vulnerable beta-lactams with 79 patients and Group 2 is further divided into AmpC-stable beta-lactams/non-beta-lactams with 33 patients. Row labels include Male sex, Age, Level of care, Hospital length of stay, Clinical signs/symptoms, Historical complicated UTI classification, and Isolated organisms of interest. Each row provides specific data points for each group and the total cohort. Notable trends include the higher percentage of male sex in Group 2 and the higher median age in Group 1. The table also shows differences in clinical signs/symptoms, historical complicated UTI classification, and isolated organisms of interest between the two groups.

Navigate back to Table 1..

## Table 2. Long description

A table comparing primary and secondary outcomes of two groups of patients treated with different antibiotics. The table has 15 rows and 5 columns. Column headers are Group 1 AmpC-vulnerable β-lactams n = 79, Group 2 AmpC-stable β-lactams/non-β-lactams n = 33, and P value (two-sided Fisher’s exact). Row labels include Primary outcome, Secondary outcomes, Change from initial antibiotics, and Antibiotic duration. Primary outcome row shows microbiological recurrence within 28 days of therapy completion with values 2 (2.53) for Group 1 and 3 (9.09) for Group 2, and P value .151. Secondary outcomes include microbiological recurrence within 365 days of therapy completion, hospitalization for recurrence, treated for recurrence, isolate with the same susceptibility pattern as prior, and 28-day recurrence with retreatment. Change from initial antibiotics includes Escalate, Broader spectrum, IV to PO, De-escalate/oral-stepdown, IV to PO, Narrower spectrum, and No change. Antibiotic duration includes Total duration of antibiotics and Total duration of active antibiotics with median (IQR) values.

Navigate back to Table 2..
