# Supplementary material for: Initial antibiotic selection and microbiological recurrence in treated bacteriuria caused by low-risk AmpC-harboring Enterobacterales: serratia marcescens, Morganella morganii, and Providencia spp
Source: Antimicrob Steward Healthc Epidemiol. 2026 Oct 8;6(1):e327. doi: 10.1017/ash.2026.10908 (PMC13760300; doi:10.1017/ash.2026.10908)
Supplement: Supplementary file 1 [file S2732494X26109085sup001.docx]

**Supplementary Table S1. Treatment transitions**

| **Supplementary Table S1. Initial-to-final regimen transitions and reported recurrence events.** | | | | | |
| --- | --- | --- | --- | --- | --- |
| **Initial** | **Change** | **Final regimen** | **N** | **Recurrence within 28 days** | **Recurrence between 28 and 365 days** |
| **Group 1 (n=79)** | | | | | |
| CRO (n=60) | No change | CRO | 23 | - | 1 |
|  | Escalation | FEP | 3 | - | - |
|  |  | FQ | 9 | - | - |
|  | De-escalation/oral stepdown | AMX/CLV | 2 | 1 | - |
|  |  | CPD | 14 | - | 1 |
|  |  | NIT | 1 | - | - |
|  |  | TMP/SMX | 8 | - | - |
| CPD (n=15) | No change | CPD | 15 | 1 | 1 |
| TZP (n=4) | No change | TZP | 1 | - | - |
|  | De-escalation/oral stepdown | CRO | 2 | - | - |
|  |  | TMP/SMX | 1 | - | - |
| **Group 2 (n=33)** | | | | | |
| FEP  (n=9) | No change | FEP | 1 | - | - |
|  | De-escalation/oral stepdown | CRO | 2 | - | - |
|  |  | FQ | 3 | - | - |
|  |  | TMP/SMX | 3 | - | 1 |
| FQ (n=11) | No change | FQ | 11 | - | - |
| MEM (n=2) | De-escalation | CRO | 1 | - | - |
|  |  | TZP | 1 | 1 | - |
| TMP/SMX (n=11) | No change | TMP/SMX | 11 | 2 | - |

Abbreviations: AMX/CLV, amoxicillin/clavulanate; CRO, ceftriaxone; CPD, cefpodoxime; FEP, cefepime; FQ, fluoroquinolones; MEM, meropenem; NIT, nitrofurantoin; TMP/SMX, trimethoprim/sulfamethoxazole; TZP, piperacillin/tazobactam.

# **Supplementary Table S2. Post hoc sensitivity analyses**

Counts are events/records; all p-values are two-sided Fisher's exact. These analyses are exploratory and unadjusted for multiplicity.

| **Restriction** | **28-day G1 vs G2** | **p** | **365-day G1 vs G2** | **p** |
| --- | --- | --- | --- | --- |
| Exclude ASB flags | 2/70 vs 3/29 | 0.148 | 3/70 vs 4/29 | 0.189 |
| Initial beta-lactam comparator | 2/79 vs 1/11 | 0.327 | 5/79 vs 2/11 | 0.203 |
| Exclude ASB + initial beta-lactam comparator | 2/70 vs 1/10 | 0.334 | 3/70 vs 2/10 | 0.115 |
| Exclude any documented FQ/TMP-SMX exposure | 2/58 vs 1/5 | 0.223 | 4/58 vs 1/5 | 0.348 |
| Confirmed simple courses confined to original class | 1/53 vs 2/24 | 0.228 | 3/53 vs 2/24 | 0.644 |
| Exclude *E. cloacae*/*C. freundii* co-pathogens | 2/77 vs 3/33 | 0.158 | 5/77 vs 4/33 | 0.448 |
